# Senescent cells: A new Achilles’ heel to exploit for cancer medicine?

**DOI:** 10.1111/acel.12875

**Published:** 2018-11-19

**Authors:** Boyi Zhang, Eric W.‐F. Lam, Yu Sun

**Affiliations:** ^1^ Key Laboratory of Tissue Microenvironment and Tumor, Shanghai Institutes for Biological Sciences University of Chinese Academy of Sciences, Chinese Academy of Sciences Shanghai China; ^2^ Department of Surgery and Cancer Imperial College London London UK; ^3^ Department of Medicine and VAPSHCS University of Washington Seattle Washington

**Keywords:** aging‐related diseases, cancer, cellular senescence, clinical trial, senescence‐associated secretory phenotype, senolytics

## Abstract

Cellular senescence is a typical tumor‐suppressive mechanism that restricts the proliferation of premalignant cells. However, mounting evidence suggests that senescent cells, which also persist *in vivo*, can promote the incidence of aging‐related disorders principally via the senescence‐associated secretory phenotype (SASP), among which cancer is particularly devastating. Despite the beneficial effects of the SASP on certain physiological events such as wound healing and tissue repair, more studies have demonstrated that senescent cells can substantially contribute to pathological conditions and accelerate disease exacerbation, particularly cancer resistance, relapse and metastasis. To limit the detrimental properties while retaining the beneficial aspects of senescent cells, research advancements that support screening, design and optimization of anti‐aging therapeutic agents are in rapid progress in the setting of prospective development of clinical strategies, which together represent a new wave of efforts to control human malignancies or mitigate degenerative complications.

1

In response to various intrinsic and/or extrinsic stimuli, cells enter an essentially irreversible senescent state, which is regulated and maintained by the p53/p21^CIP1^ and p16^INK4a^/pRB pathways to prevent the occurrence of sporadic events, particularly transformation. Senescent cells display several distinct features including a flattened and enlarged morphology, DNA segments with chromatin alterations reinforcing senescence (DNA SCARS), nuclear heterochromatin foci and senescence‐associated β‐galactosidase (SA‐β‐Gal) activity (Ozcan et al., 2016). However, senescent cells are frequently implicated in multiple disorders, mainly through secretion of numerous bioactive molecules, a distinctive phenomenon found a decade ago and termed as the senescence‐associated secretory phenotype (SASP; Acosta et al., 2008; Coppe et al., 2008; Kuilman et al., 2008). The full SASP spectrum comprises a myriad of soluble factors including pro‐inflammatory cytokines, chemokines, growth factors and proteases, whose functional involvement can be classified into several aspects including but not limited to extracellular matrix formation, metabolic processes, ox‐redox events and gene expression regulation (Ozcan et al., 2016). The SASP promotes embryonic development, tissue repair and wound healing, serving as an evolutionarily adapted mechanism in maintaining tissue and/or organ homeostasis (Davaapil, Brockes, & Yun, 2017; Demaria et al., 2014; Jun & Lau, 2010; Munoz‐Espin et al., 2013; Storer et al., 2013). Senescent cells communicate with their surrounding environment by expressing the SASP, with the potential to boost immune surveillance by mounting specific inflammatory responses including those mediated by CD4^+^ T cells against antigens expressed in senescent cells, particularly those observed in premalignant lesions (Georgilis et al., 2018; Kang et al., 2011; Toso et al., 2014). Although the SASP is beneficial to several health‐associated events, more evidence has showed that it actively contributes to the formation of a pro‐carcinogenic tumor microenvironment (TME). Long‐term secretion of the SASP factors by senescent cells can impair the functional integrity of adjacent normal cells in the local tissue, serving as a major cause of chronic inflammation which drives aging‐related degeneration of multiple organs (He & Sharpless, 2017). Thus, senescent cells and their unique phenotype, the SASP, can be defined as a form of antagonistic pleiotropy, a property that is beneficial in early life and during tissue turnover, but deleterious over time with advanced age, making both mechanistic investigation and therapeutic intervention of paramount significance in current era of precision medicine.

As the SASP can generate contrasting pathophysiological consequences, substantial interest has been sparked in recent years to achieve an accurate and thorough understanding of this cell‐non‐autonomous phenotype. In cancer patients, the most frequently observed formats of cellular senescence encompass oncogene‐induced senescence (OIS) and therapy‐induced senescence (TIS) (Sieben, Sturmlechner, Sluis, & Deursen, 2018) (Figure 1). Indeed, both modalities are initially tumor suppressive, but later tend to manifest a pro‐tumorigenic capacity by substantially activating the DNA damage response (DDR), which once perceived irreparable by the damaged cells can potently induce the SASP (Rodier et al., 2011). It is now clear that regulation of the initiation and development of the SASP involves multiple signaling pathways, including those mediated by p38MAPK, Jak2/Stat3, the inflammasome, mTOR, GATA4, macroH2A1, ATM and mitochondrial sirtuins (Ito, Hoare, & Narita, 2017). Although some SASP effectors appear to act post transcriptionally, most SASP regulators converge on two transcription factors, NF‐кB and C/EBPβ, which co‐regulate many SASP components (Di Mitri & Alimonti, 2016). Furthermore, some interleukins (ILs) are encoded by the SASP but can reciprocally modulate the SASP by feedback mechanisms, such as IL6, IL‐8 and IL‐1α (Di Mitri & Alimonti, 2016). Although activation of DNA damage response (DDR) is essential for the induction and maintenance of senescence (Rodier et al., 2009, 2011 ), the precise regulatory mechanism directly linking the DDR events to the SASP development remains largely unclear until emergence of recent data, which revealed the implication of a cGAS‐STING (cGMP‐AMP synthase‐stimulator of interferon genes) pathway. Briefly, cGAS is a highly conserved cytosolic DNA sensor, which can be activated once bound by double‐stranded DNA released from genome instability‐induced micronuclei, a process that engages a second messenger cGMP‐AMP (cGAMP), which subsequently triggers the adaptor protein STING to recruit TANK‐binding kinase 1 (TBK1) and IκB kinase to activate IFN regulatory factor 3 (IRF3) and NF‐κB, respectively, causing the production of type I interferons and expression of numerous SASP factors (Dou et al., 2017; Gluck et al., 2017; Mackenzie et al., 2017; Yang, Wang, Ren, Chen, & Chen, 2017). However, how the cGAS‐STING pathway is functionally connected with other SASP modulators including but not limited to p38MAPK, Jak2/Stat3 and GATA4, remains an open question that merits future exploration. Given the remarkable complexity of the SASP signaling, further experimental inputs are essential to achieve new insights and to present optimal molecules for therapeutic targeting of such a distinctive phenotype.

**Figure 1:**
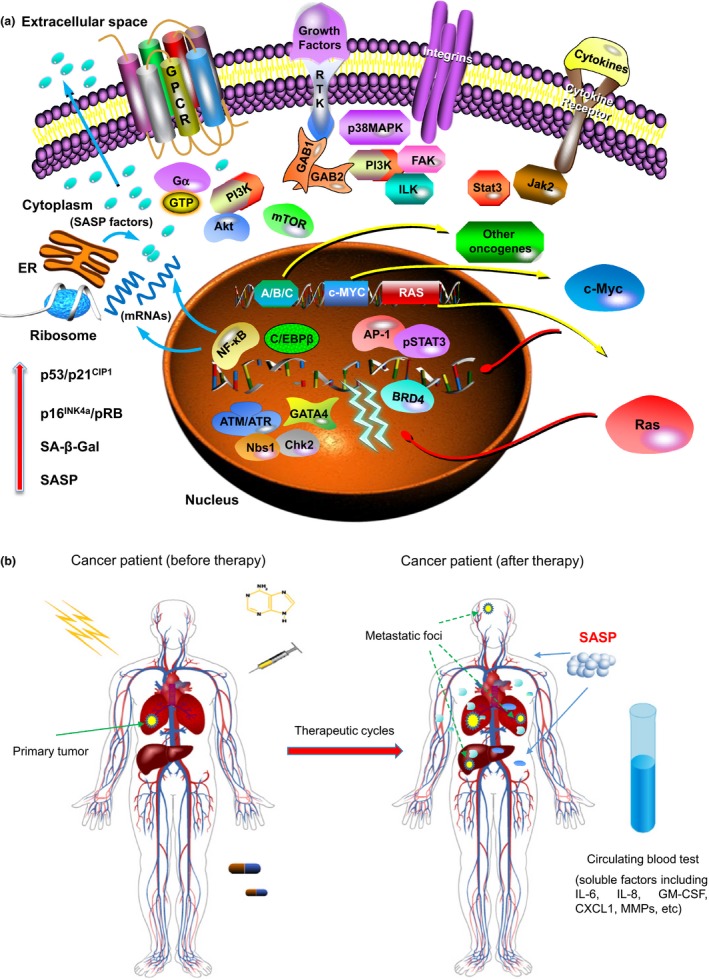
Oncogene‐ and therapy‐induced cellular senescence. (a) oncogene‐induced senescence (OIS) represents a cell responsive program provoked upon aberrant activation of specific oncogenes such as Ras, Raf, Akt, Cyclin E and c‐Myc (Acosta & Gil, 2012; Ko et al., 2018; Warnier et al., 2018). OIS results from the enforcement of a DDR triggered by DNA hyper‐replication induced by oncogene expression, a process that is initially transient but ultimately ends with the permanent establishment of cellular senescence (Di Micco et al., 2006). In such a case, persistent DDR events are observed in senescent cells, and molecules such as ATM/ATR, Nbs1 and Chk2 are actively engaged in DDR‐associated signaling. Regulation of the SASP is subject to multiple intracellular pathways including but not limited to p38MAPK, Jak2/Stat3 and mTOR (Freund, Patil, & Campisi, 2011; Laberge et al., 2015; Toso et al., 2014), which inevitably converge on transcription factors such as NF‐кB, C/EBPβ and AP‐1 (Han et al., 2018; Ito et al., 2017). Recent studies revealed that GATA4 is an upstream modulator of NF‐кB signaling in senescent cells, while the chromatin reader protein BRD4 dynamically binds to super‐enhancer elements adjacent to the genes encoding SASP factors (Kang et al., 2015; Tasdemir et al., 2016). As different cell types show different responses to oncogenic stress, the relevant mechanisms dictating the sensitivity or resistance to a specific oncogene remain to be elucidated by future investigations. (b) Therapy‐induced senescence (TIS) can be typically induced in normal, immortal or transformed, and cancer cells by anticancer compounds or ionizing radiation. Although generally considered tumor suppressive, TIS has recently been demonstrated by multiple studies to be able to enhance cancer resistance, relapse and metastasis by causing diverse cytotoxicity‐related side effects including an in vivo form of the SASP (Chen et al., 2018; Kim et al., 2017; Mikula‐Pietrasik et al., 2016; Wieland et al., 2017; Zhang et al., 2018). Furthermore, experimental data suggested that TIS induced by genotoxic chemotherapy promotes cancer metastasis from primary sites to distant organs (Demaria et al., 2017). It is imaginable that similar consequences could be observed in cancer clinics, a process driven by senescent cells with the tendency to promote malignant progression in the post‐treatment stage, particularly cancer metastasis. We also raise the possibility of assaying typical SASP factors in peripheral blood of cancer patients for appraisal of treatment outcome and prognosis of disease exacerbation, a significant and innovative strategy of the SASP‐based pathological assessment that may be realized in future medicine. Abbreviations and notes: A/B/C, oncogenes alternative to those exemplified (c‐Myc and Ras) in (a); ER, endoplasmic reticulum; SASP, senescence‐associated secretory phenotype; SA‐β‐Gal, senescence‐associated β galactosidase; DDR, DNA damage response; TME, tumor microenvironment; GATA4, GATA binding protein 4; BRD4, bromodomain containing 4; dashed lines in (b), potential metastatic sites of disseminating cancer cells driven by the impact of TIS in patients that have undergone anticancer therapy

In clinical medicine, anticancer agents not only triggers significant apoptosis of cancer cells but also causes substantial damage in the TME and induces typical TIS of the resident stromal cells, which cause therapeutic resistance via secretion of the SASP factors (Chen et al., 2018; Sun et al., 2012, 2016 ). Interestingly, damage‐provoked SASP can also be restrained to preserve tissue homeostasis and prevent chronic inflammation, as suggested by recent study that revealed PI3K/Akt/mTOR pathway as a molecular rheostat to control the SASP progression (Bent, Gilbert, & Hemann, 2016). Indeed, a TME‐specific stress response is engaged promptly upon cellular damage particularly those induced by genotoxic insults, and stromal cells exhibit an acute stress‐associated phenotype (ASAP) characterized by subsequent secretion of a small handful of soluble factors including IL‐6 and Timp 1 (Gilbert & Hemann, 2010). In contrast to the ASAP as a rapid response mainly involving the ATM‐TRAF6‐TAK1 axis, the SASP is a relatively chronic process governed by key signaling nodes such as TAK1, a central kinase that functionally mediates phenotypic transition from the ASAP to the SASP and holds remarkable potential as an optimal therapeutic target to manipulate the SASP with a higher efficacy than that of p38‐ or mTOR‐oriented suppression (Zhang et al., 2018).

A new function of the SASP was recently discovered, which is linked with increased expression of stem cell markers and keratinocyte plasticity upon short term exposure of cells to the SASP *in vitro* and liver regeneration of a treatment‐inducible OIS mouse model *in vivo*, thus raising the possibility that transient therapeutic delivery of senescent cells could be harnessed to promote tissue regeneration (Ritschka et al., 2017). Interestingly, another study used agent‐inducible senescence animal models targeting trimethylation of lysine 9 at histone H3 (H3K9me3) or p53 to simulate spontaneous escape from cellular senescence, and found that cells released from senescence can re‐enter cell cycle with pronouncedly enhanced stemness and Wnt‐dependent growth potential compared to identical cell populations exposed to same chemotherapy but without experiencing senescence (Milanovic et al., 2018). Thus, senescence‐associated reprogramming promotes cancer stemness (senescence‐associated stemness, or SAS), a distinct property that has profound implications for cancer therapy and presents new mechanistic insights into cancer cell plasticity. Partially resembling cancer cells which pose substantial threat to human lifespan, senescent cells are functionally involved in tumor progression and can be viable targets for some reasons. First, senescent cells share common biochemical features, allowing use of a single therapeutic agent to eliminate them from the tissue microenvironment. Second, new protocols targeting senescent cells could practically synergize with hitherto established or proposed anticancer programs, which are frequently based on a presenescence scenario (Acosta & Gil, 2012). Given that many chemotherapeutics induces collateral senescence in the TME, pharmaceutical agents targeting senescent cells can be a key component of advanced anticancer arsenal (Childs et al., 2017). However, is there a way to radically remove senescent cells in the damaged or aged tissue rather than merely inhibition of the SASP, so that long‐term drug administration can be circumvented?

Several recent studies provided a series of pilot evidence in specific clearing senescent cells, including single or dual treatment of senescent cells with quercetin/dasatinib, and pan‐BCL inhibition with ABT‐263/ABT‐737 (Chang et al., 2016; Yosef et al., 2016; Zhu et al., 2015). Frequently detected in fruits and vegetables, quercetin is natural product and beneficial against aging, as evidenced by its capacity in attenuating premature senescence of human mesenchymal stem cells (hMSCs) in Hutchinson‐Gilford progeria syndrome and postponing physiological‐aging of hMSCs in Werner syndrome (Geng et al., 2018). Dasatinib is a suppressor of Src kinase family and has showed prominent efficacy for some cancer types including chronic myeloid leukemia and colon cancer (Benthani et al., 2018; Naqvi et al., 2018). The senolytic cocktail consisting of dasatinib and quercetin reduces the number of naturally occurring senescent cells in explants of human adipose tissue, while intermittent oral administration of senolytics to either senescent cell‐implanted young animals or naturally aged mice can alleviate physical dysfunction and extend post‐treatment survival (Xu et al., 2018). However, both compounds (dasatinib and quercetin) were considered to be nonspecific among types of senescent cells and can display cell type‐dependent effects (Zhu et al., 2015). Given the prominent efficacy of the senolytic cocktail in controlling aging‐related symptoms as demonstrated in multiple lines of experimental mice including those of an immunodeficient or immunocompetent background, and in human adipose tissue explants (Ogrodnik et al., 2017; Schafer et al., 2017; Xu et al., 2018), a comprehensive and practical use of these compounds as clinical senolytics upon systemic evaluation is intriguing for future medicine. In addition, BCL inhibitors or BH3 mimetic drugs appeared to be an alternative group of agents against senescent cells by specifically targeting multiple BCL family members including BCl‐2, BCL‐xl, and BCL‐w (Chang et al., 2016; Yosef et al., 2016). Specifically, ABT263 (also navitoclax) reduces viability of senescent human lung fibroblasts (IMR90), human umbilical vein epithelial cells (HUVECs) and murine embryonic fibroblasts (MEFs), but not human primary preadipocytes, thus is senolytic in some, but not all types of senescent cells (Zhu et al., 2016). ABT263 has been extensively applied with success in treatment of human malignancies including lymphoma and multiple solid tumors; while another BCL inhibitor ABT737 has experienced an *ex vivo* evaluation in ovarian tumor samples (Lheureux et al., 2015). Unfortunately, a major drawback of BCL‐targeting agents merits attention, which predominantly results from their pronounced cytotoxicity, especially BCL‐2 inhibitors such as ABT263 and ABT199 (also venetoclax), the strong apoptosis inducers that pose a substantial risk to most cell types. Although applicable for immediate life‐threatening conditions including advanced malignancies, off‐target damage should be avoided intentionally for cancer patients and those at high age. Despite the antisenescence potential of these agents, future studies should be able to address whether further optimization is technically feasible or more selective agents can be designed, the latter ideally targeting intracellular molecules/pathways that are specifically up‐ or down‐regulated in senescent cells and are inherently correlated with their survival. To date, several senolytic molecules have been identified that show promising potency and selectivity such as a D‐retro inverso (DRI) peptide that perturbs FOXO4 interaction with p53 and causes pronounced apoptosis of senescent cells (Baar et al., 2017). Furthermore, utilization of these “first‐generation” senolytic strategies in preclinical models is disease‐minimizing, presumably through attenuation of the SASP. This implies that diseases associated with senescent cells, such as cancer, may be amenable to senotherapy mediated by agents that are in currently ongoing clinical trials but have the potential to be exploited as modulators or eliminators of senescent cells (Table 1).

**Table 1:** Small molecule agents that hold potential as SASP inhibitors or senolytics in cancer clinics

Agent	Target (s)		Target class	Development status	References
ABT‐263	BCL‐2/BCL‐XL	Pro‐survival or anti‐apoptotic factors		Preclinical animal models/Clinical trials (phase I/II (NCT00406809 for leukemia and lymphoma/NCT00445198 for lung cancer), phase I (NCT00743028 for leukemia and lymphoma/NCT00982566 for lymphoma and solid tumors), and phase II (NCT02591095 for ovarian cancer/NCT01557777 for leukemia))	Chang et al. (2016)
ABT‐737	BCL‐w/BCL‐XL	Pro‐survival or anti‐apoptotic factors		Preclinical animal models/Ex vivo evaluation of ovarian tumor (NCT01440504)	Yosef et al. (2016)
Dasatinib	Pan‐receptor tyrosine kinases	Receptor tyrosine kinases		Clinical trials (Phase I/II (NCT00597038 for melanoma/NCT00550615 for lymphoma), Phase I (NCT00652574 for mesothelioma/NCT01744652 for advanced cancers), Phase II (NCT02744768 for leukemia/NCT00429949 for myeloma), Phase III (NCT02013648 for leukemia), Phase IV (NCT03216070 for leukemia))	Xu et al. (2018) and Zhu et al. (2015)
Metformin	The IKK complex and/or NF‐κB	The SASP		Approved for type II diabetes/Clinical trials for cancer (Phase I/II (NCT02949700 for head and neck squamous cell carcinoma), Phase II (NCT03137186 for prostate cancer/NCT03398824 for Fanconi Anemia/NCT02506777 for breast cancer)), clinical trials for aging (Phase IV (NCT02745886 for aging/NCT02432287 for aging))	Oubaha et al. (2016)
Rapamycin	Mechanistic target of rapamycin kinase (mTOR)	The SASP		Approved for immunosuppression/Clinical trials for cancer (Phase I (NCT02724332 for liver cancer/NCT03014297 for neuroendorine tumors))	Herranz et al. (2015) and Laberge et al. (2015)
RAD001	Mechanistic target of rapamycin kinase (mTOR)	The SASP		Approved for immunosuppression, clinical trials for cancer (Phase I/II (NCT00516165 for liver cancer/)， Phase II (NCT00782626 for glioma and astrocytoma/NCT01051791 for head and neck squamous cell carcinoma/NCT01152840 for adenoid cystic cancer))	Zhang et al. (2018)
LY2228820	p38MAPK	The SASP		Clinical trials for cancer (Phase I (NCT01393990 for advanced cancer), Phase I/II (NCT01663857 for ovarian cancer, NCT02364206 for glioblastoma)	Freund et al. (2011)
LY3007113	fp38MAPK	The SASP		Clinical trials for cancer (Phase I (NCT01463631 for advanced cancer))	Freund et al. (2011)
Quercetin	Lipoprotein lipase (LPL) and potassium voltage‐gated channel subfamily E regulatory subunit 2 (KCNE2)	Antioxidant enzymes		Phase II clinical trial (NCT02848131) for chronic kidney disease	Zhu et al. (2015)
FOXO4‐DRI	Interaction between FOXO4 and p53	Pro‐survival or anti‐apoptotic factors		Preclinical animal models	Baar et al, (2017)
5Z‐7‐Oxozeaenol	Transforming growth factor‐β1‐activated kinase‐1 (TAK1)	The SASP		Preclinical animal models	Zhang et al, (2018)

Cellular senescence occurs throughout lifespan, and senescent cells are beneficial to certain physiological and pathological processes including embryonic patterning, tissue repair, wound healing and immune surveillance. However, as address above, a steady accumulation of senescent cells in the tissue has adverse consequences, ultimately enhancing clinical morbidity. Thus, the abundance of senescent cells *in vivo* may serve as a “molecular” marker for disease occurrence and guide patient stratification (Demaria et al., 2017), a novel approach for clinical advancement which can be correlated with benefit of senotherapy.

Despite all the recent findings from senescence and cancer research, there are several caveats before we move forward. Agents targeting senescent cells, especially SASP inhibitors, should be investigated meticulously to ensure continued maintenance of cell cycle arrest, as bypassing the crisis can inevitably promote carcinogenesis. As senescent cells also have certain health‐promoting functions, identification of the beneficial components of the SASP could lead to development of optimal strategies that preserve vital factors while depleting their detrimental counterparts derived from senescent cells. As a technical issue, achieving the balance between deleterious and beneficial impact of senolytics in cancer patients requires careful and rational design of administration regimens such as classic chemotherapy followed by senolytic treatment, each provided in metronomic cycles to minimize *in vivo* toxicity but enhancing overall efficacy. Such a therapeutic modality is desirable and holds the potential to enhance patient treatment efficacy while reducing adverse side effects that can be observed upon administration of each agent in a single dose. Finally, targeting senescent cells while simultaneously promoting tissue regeneration represents an optimal solution to remove senescent cells from individuals particularly those with advanced diseases or at later stage in life. In doing so, we are getting even closer to achieving the goal of a real “healthy” therapy against human cancer and aging.

## CONFLICT OF INTEREST

None Declared.
